# The Conventional Non-Articulated SACH or a Multiaxial Prosthetic Foot for Hypomobile Transtibial Amputees? A Clinical Comparison on Mobility, Balance, and Quality of Life

**DOI:** 10.1155/2015/261801

**Published:** 2015-05-11

**Authors:** Francesco Paradisi, Anna Sofia Delussu, Stefano Brunelli, Marco Iosa, Roberto Pellegrini, Daniele Zenardi, Marco Traballesi

**Affiliations:** ^1^Santa Lucia Foundation, Scientific Institute for Research, Hospitalization and Health Care, Via Ardeatina 306, 00179 Rome, Italy; ^2^Department of Movement, Human and Health Sciences, Interuniversity Centre of Bioengineering of the Human Neuromusculoskeletal System, University of Rome “Foro Italico”, Piazza Lauro De Bosis 6, 00135 Rome, Italy; ^3^ITOP SpA Officine Ortopediche, Via Prenestina Nuova 163, Palestrina, 00036 Rome, Italy

## Abstract

The effects of a non-articulated SACH and a multiaxial foot-ankle mechanism on the performance of low-activity users are of great interest for practitioners in amputee rehabilitation. The aim of this study is to compare these two prosthetic feet and assess possible improvements introduced by the increased degrees of freedom provided by the multiaxial foot. For this purpose, a group of 20 hypomobile transtibial amputees (TTAs) had their usual SACH replaced with a multiaxial foot. Participants' functional mobility, involving ambulatory skills in overground level walking, ramps, and stairs, was evaluated by performing Six-Minute Walking Test (6MWT), Locomotor Capability Index-5 (LCI-5), Hill Assessment Index (HAI), and Stair Assessment Index (SAI). Balance performances were assessed using Berg Balance Scale (BBS) and analysing upper body accelerations during gait. Moreover, the Prosthesis Evaluation Questionnaire (PEQ) was performed to indicate the prosthesis-related quality of life. Results showed that participants walked faster using the multiaxial foot (*p* < 0.05) maintaining the same upright gait stability. Significant improvements with the multiaxial foot were also observed in BBS, LCI-5, and SAI times and 4 of 9 subscales of the PEQ. Our findings demonstrate that a multiaxial foot represents a considerable alternative solution with respect to the conventional SACH in the prosthetic prescription for hypomobile TTAs.

## 1. Introduction

Prosthetic feet are devices designed to replace one or more function of the biological human ankle-foot system. Over the past few years developers have released to the market a large variety of technologically advanced prosthetic feet, broadening the range of available devices.

Despite the technological progress, the selection of the most appropriate foot for each person with amputation is still difficult. Anecdotal evidence indicates that prescription of optimal prosthetic feet to ensure successful rehabilitation is challenging because there are no generally accepted clinical guidelines based on objective data [1]. Indeed, despite depending on the patient's Medicare Functional Classification Level (MFCL) [2], the proper choice is often derived from the particular clinical experience of the prosthetist and the rehabilitation team [3]. Translational research in this field could provide the scientific evidence required to improve and make more objective the prescriptions of prosthetic feet to persons with lower limb amputation.

Most studies concerning the influence of prosthetic feet in transtibial amputees (TTAs) on gait compared one or more energy storing and return (ESAR) devices and the conventional and most frequently studied Solid Ankle Cushioned Heel (SACH) foot [4]. ESAR feet used by active TTAs were shown to provide better clinical effects than the SACH foot, in terms of energy cost of walking [5], gait symmetry in ascending stairs [6], and biomechanical parameters such as increased ankle range of motion (RoM) and power absorption in prosthetic ankle during weight bearing [7]. Conversely, the SACH is considered to be the most appropriate foot for hypomobile TTAs and also the most prescribed, as it is inexpensive, easy to use, and perceived as stable and safe by hypomobile amputees [8–10]. However, the SACH has many disadvantages; for instance, the time between heel strike and foot flat is twice as long as that in normal gait [11]; it almost never remains flat during the stance phase and the heel rises very early, which makes it less versatile on uneven terrain [10]. Moreover walking on inclines with the SACH is particularly demanding, especially in descending ramps [12]. As ESAR feet are not recommended for hypomobile TTA [10] due to the perceived instability of energy return, an alternative to the conventional SACH could be represented by multiaxial feet. Indeed, multiaxial feet have no energy returning capacity while allowing higher degrees of freedom (DoFs) and RoM at the joint, therefore providing a closer representation of a natural foot-ankle system than the SACH. Despite its potential, this type of foot has not been studied thoroughly yet.

Actually only two studies have compared the SACH with a multiaxial foot [1, 13]. Marinakis [13] found significant improvement in spatial-temporal parameters, symmetry index of the hip, and ankle RoM when SACH was replaced by the Greissinger Plus foot, composed of a rigid longitudinal keel and a multiaxial ankle. However, participants were not confident prosthetic users but only in the early stages of rehabilitation. Moreover the study did not include structured questionnaires that cover major aspects of everyday life that might be affected by living with prosthesis. The second study [1] presents a comparison between ESAR and SACH feet, both with and without a multiaxial joint. The authors showed that older low-mobility TTAs did not benefit from the ESAR but did benefit from the increased flexibility provided by multiaxial ankles. In fact, the majority of participants (11 of 15) preferred the SACH foot integrated with the multiaxial joint (called SACH-MA), designed specifically for the study but not available commercially. However, the authors concluded that the results could provide a scientific rationale for prescribing a multiaxial ankle to improve TTAs gait performance.

In the light of the above, a clinical comparison between SACH and a multiaxial prosthetic foot for low-mobility TTAs is both useful and necessary in order to allow practitioners to prescribe the most adequate device. The hypothesis underlying this study is that the use of a multiaxial foot allows an improved performance in confident SACH users TTAs, as it presents more DoFs without introducing other elements that may jeopardise the user's perceived safety.

Thus the main aim of this study is to compare the clinical effects of SACH and multiaxial prosthetic feet on mobility, balance performances, and prosthesis-related quality of life in hypomobile unilateral TTAs.

## 2. Materials and Methods

### 2.1. Subjects

All of the consecutive TTAs treated in the outpatients department of our rehabilitation hospital were screened for the following enrolling criteria: (a) body mass < 125 kg, (b) functional mobility K-level 1 (the patient has the ability or potential to use a prosthesis for transfers or ambulation on level surfaces at fixed cadence: this is typical of a household ambulator or a person who only walks about in their own home) or K-level 2 (the patient has the ability or potential for ambulation with the ability to traverse low level environmental barriers such as curbs, stairs, or uneven surfaces: this is typical of the limited community ambulator) [2], (c) SACH foot users for at least 6 months and for a minimum of 4 hours per day, and (d) absence of severe comorbidities or clinical residual limb complications.

### 2.2. Prosthetic Feet

**Figure 1:**
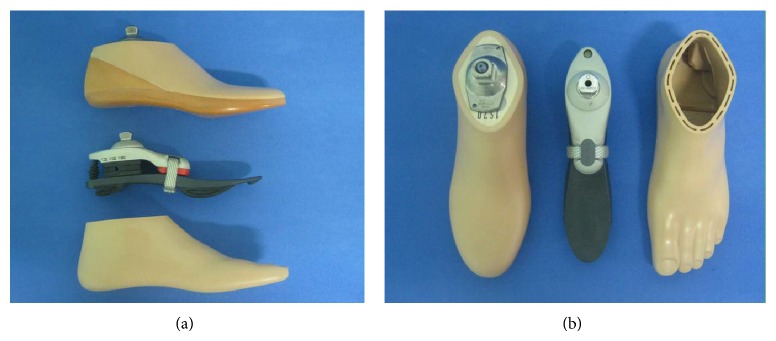
The SACH and the 1M10 Adjust prosthetic feet on the sagittal (a) and the transversal (b) plane. The SACH foot is placed on the top in (a) and on the left in (b), while the 1M10 Adjust is depicted with the respective cosmetic cover.

Two types of prosthetic feet were used (Figure 1), the SACH and the 1M10 Adjust (1M10), both manufactured by Otto Bock HealthCare GmbH (Duderstadt, Germany).

SACH has no ankle joint mechanism. It is composed of a rigid wooden keel that provides midstance stability and forms the internal component. The keel is covered by plastic polymers with different densities that supply the function of cushioning the heel strike (cushioned heel) and facilitating the forefoot rocker, while maintaining the ankle stiffness (solid ankle).

1M10 is a prosthetic foot with multiaxial joint positioned at load line, made up of a flexible functional module and a forefoot ball-pad that could help the users' stability during walking and standing, as claimed by the manufacturer. Multiaxial behaviour permits inversion-eversion on the frontal and dorsiplantar flexion on the sagittal plane.

### 2.3. Outcome Measures and Tools

To thoroughly investigate the three clinical aspects of interest we chose the following outcome measures. Mobility was assessed by the patient's ambulatory skills on floor, ramps, and stairs by means of the Locomotor Capability Index-5 (LCI-5) [14], Six-Minute Walking Test (6MWT) [15], Hill Assessment Index (HAI) [16], and Stairs assessment Index (SAI) [16]. Balance was assessed by means of the Berg Balance Scale (BBS) [17] and gait stability through analysis of upper body accelerations. Finally we took into consideration the feedback of participants concerning quality of life and general comfort perceived with each foot by means of the self-report Prosthesis Evaluation Questionnaire (PEQ) [18].

#### 2.3.1. Locomotor Capability Index-5 (LCI-5)

The LCI-5 [14] evaluates the subject's ambulatory skills through the assessment of the subject's capability performing 14 different activities while wearing prosthesis, rated with a 5-point ordinal scale ranging from 0 to 4. The maximum total score of the index is 56. The self-report measure of LCI-5 has demonstrated good test-retest reliability and internal construct validity in elderly and middle-aged persons with amputation [14].

#### 2.3.2. Six-Minute Walking Test (6MWT)

The 6MWT [15] consists of walking for 6 minutes at one's own self-selected walking speed (SSWS). It is a reliable and useful measure of functional mobility especially for people with impairment [19], TTAs included [20]. In studies of prosthetic components SSWS has often been used as a parameter for the quality of performance [21, 22] also when different prosthetic feet were compared [23]. According to other studies [24] the average SSWS (m/s) for each participant was computed dividing the covered distance by 360 seconds.

#### 2.3.3. Hill Assessment Index (HAI) and Stair Assessment Index (SAI)

The HAI [16] and the SAI [16] evaluate the ability to walk up/down on an inclined surface and to ascend/descend a certain number of stair steps, respectively. Both of these performance-based tests were used in previous works to evaluate or compare different prosthetic components and feet [25, 26]. Also the time (s) required in HAI going uphill/downhill and SAI walking up/down at self-selected speeds was recorded as previously reported by Highsmith et al. [27]. The stair used for SAI was 2 meters wide and had 12 steps, 31 centimeters deep and 18 centimeters high, with a handrail on the right side. The ramp used for HAI was 28 meters long and paved with a slope of 10 degrees.

#### 2.3.4. Berg Balance Scale (BBS)

The BBS [16] is composed of 14 functional tasks, each of them ranking 0–4; the maximum total score of the index is 56. Major et al. [28] demonstrated the high BBS validity and reliability for assessing balance in lower limb amputees.

#### 2.3.5. Upright Gait Stability (UGS)

**Figure 2:**
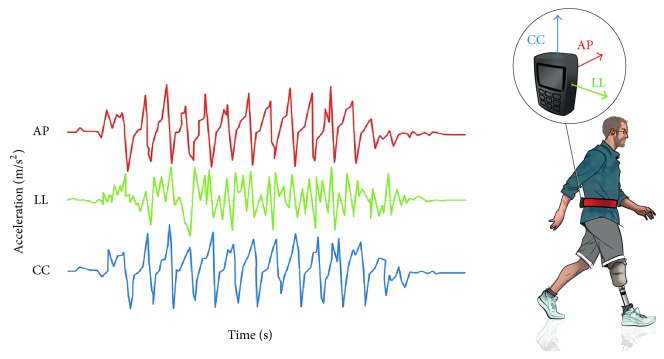
The Upright Gait Stability assessment during walking. The triaxial accelerometer placed on the back area (L2-L3) of each subject allows expressing the acceleration's signals along the three anatomical axes: anteroposterior (AP, red line), laterolateral (LL, green line), and craniocaudal (CC, blue line).

The UGS has been defined as the capacity to minimize upper body oscillations and absorb jerks, bumps, shakes, and fluctuations, despite the broad and fast movements of the lower limbs during locomotion [29, 30]. Hence, an upright gait is stable when upper body accelerations are minimized and smoothed [31]. The methodological approach followed in this work was similar to many previous studies concerning other pathological populations and already used also in amputees [32]. In detail, each person was equipped with an Inertial Measurement Unit (IMU) (FreeSense, Sensorize srl; Rome, Italy) during the 6MWT (Figure 2). This small and lightweight device was placed within an elastic belt worn by patients and located on an area of their back corresponding to the L2-L3 spinous processes [33]. The considered IMU contains a triaxial accelerometer and two biaxial gyroscopes and provides data with respect to a local sensor-embedded frame coinciding with the geometrical axis of the IMU case. The device allows expressing both acceleration and angular velocity signals along the three anatomical axes: anteroposterior (AP), laterolateral (LL), and craniocaudal (CC). Sampling frequency at 100 frames/s was used. The IMU data extraction and analysis were performed with MATLAB (version 8.2).

The three accelerometric signals were low-pass filtered using a 20 Hz low-pass 2nd order Butterworth filter applied to the signals after their mean value subtraction. The Root Mean Square (RMS) of acceleration was then computed, as measure of acceleration dispersion that coincides with the standard deviation because of the signal mean subtraction. As the RMS of acceleration is strictly dependent on walking speed, the values of RMS-AP and RMS-LL were normalized with respect to those of RMS-CC by using the inverse of their percentage ratio as an indicator of UGS. These normalized parameters were shown to be suitable for assessing the trunk stability during walking both in persons with stroke [31] and amputation [32], with higher values of normalized RMS representing higher instabilities.

#### 2.3.6. Prosthesis Evaluation Questionnaire (PEQ)

Users' satisfaction with the prosthesis was assessed by means of the PEQ [18]. The questionnaire consists of a series of items with a linear analogical scale response format, organized into nine functional domain scales, widely used to evaluate the effects on TTAs prosthesis-related quality of life with different prosthetic feet [26, 34]. The functional scales are ambulation, appearance, frustration, perceived response, residual limb health, social burden, sounds, utility, and well-being. The reliability and validity of this survey have previously been assessed and approved [35, 36].

### 2.4. Data Collection

**Figure 3:**
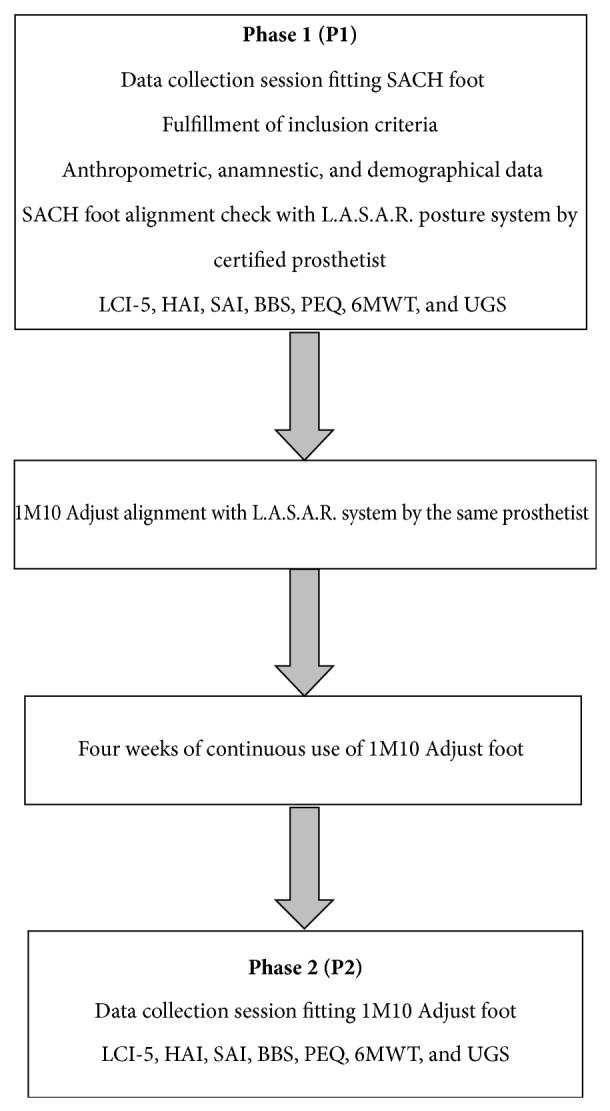
Study design and timing. The L.A.S.A.R. posture system was used to check the alignment of SACH and to optimize that of the 1M10 Adjust by the same certified prosthetist. Outcome measures of Locomotor Capability Index-5 (LCI-5), Hill Assessment Index (HAI), Stair Assessment Index (SAI), Berg Balance Scale (BBS), Prosthesis Evaluation Questionnaire (PEQ), Six-Minute Walking Test (6MWT), and Upright Gait Stability (UGS) were collected fitting SACH during the first phase of evaluation (P1) and 1M10 Adjust foot after one month of acclimation (P2).

The study was organized into two main phases of evaluation. During the first phase (P1) fulfillment of inclusion criteria was verified; anthropometric, anamnestic, and demographical information was also collected. The same physiatrist, experienced in amputee rehabilitation, classified each person enrolled according to their functional level with the MFCL system. Then a certified prosthetist verified the proper SACH alignment with respect to a reference line by means of a posture system (L.A.S.A.R. Posture, Otto Bock HealthCare GmbH, Duderstadt, Germany). If the alignment did not appear to be correct, the patient was automatically excluded from the study. Later all self-report and performance-based tests described above were administrated. Participants carry out the performed tests (i.e., 6MWT, UGS, BBS, HAI, and SAI) during a morning session, allowing enough recovery time between each test in order to avoid effect of fatigue on measurements. Note that UGS data are recorded simultaneously to 6MWT. At the end of P1 the same prosthetist changed the SACH with the 1M10 without changing the socket. A correct alignment was enured with the same posture system. Participants continuously used 1M10 for 4 weeks to ensure adequate prosthesis acclimation [37] and were required to maintain their lifestyle unchanged (i.e., physical activity, nutritional regimen, etc.) for the entire time of the study. In the second phase of evaluation (P2) all participants performed the same tests as P1 fitting the multiaxial foot. The same researcher administered each outcome measure in both data collection sessions. Each performance-based test was carried out once to avoid learning effects. The experimental tests in P1 and P2 were conducted with the same pair of shoes and at the same hour of the morning to avoid inaccurate data due to eventual fluctuations in residual limb volume. A flowchart of the study design is shown in Figure 3.

The local ethics committee approved the study procedures that adhered to the Declaration of Helsinki for medical research involving human subjects for the study, and informed consent was obtained from all subjects prior to their participation on a voluntary basis.

### 2.5. Statistical Analysis

Statistical analyses were conducted using SPSS (version 17.0 for Windows). Nonparametric statistics were used for ordinal scores of clinical scales (BBS, LCI-5, HAI, and SAI) and questionnaire (PEQ). In particular these ordinal data have been described using median, quartiles, and interquartile range (i.e., the difference between third and first quartiles) and compared between P1 and P2 using Wilcoxon signed rank test. Parametric statistics were used for continuous quantitative measures such as the measured time of HAI and SAI tests, the self-selected walking speed in 6MWT, and the root mean squares of trunk accelerations. These continuous data have been reported in terms of means and standard deviations and compared between P1 and P2 using Student's *t*-test. As the RMS of acceleration is strictly dependent on the walking speed, the values of RMS-AP and RMS-LL were normalized with respect to those of RMS-CC, analogous to previous studies [30, 31]. The critical alpha was set at 0.05 for all data analyses.

## 3. Results

Twenty-one subjects were enrolled in this study, of those, one dropped out for heart disease. Clinical and demographical data are reported in Table 1.

Concerning the clinical outcome score results, statistically significant improvements were found at P2 in the LCI-5, BBS, HAI, and SAI scores (Table 2).

Significant differences were found both in SAI going up and down times, whereas none were found in HAI uphill and downhill times (Table 3).

**Figure 4:**
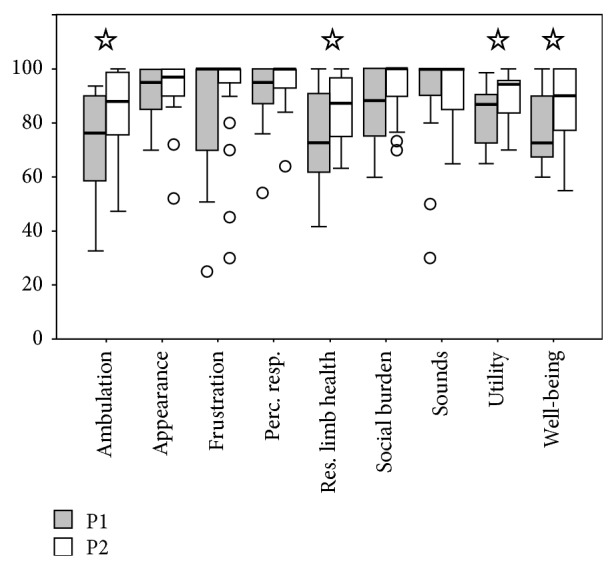
Box-plots for the nine subscales scores of the Prosthesis Evaluation Questionnaire (PEQ). The boxes show the lower quartile, median (bold line), and upper quartile values, the whiskers represent the most extreme values within 1.5 times the interquartile range from the ends of the box, and the circles represent the outliers (data with values beyond the ends of the whiskers). The ordinal data of the nine functional scales of PEQ have been compared between P1 and P2 using Wilcoxon signed rank test.

The PEQ values showed significant improvements in these domains: ambulation, residual limb health, utility, and well-being (Figure 4).

The mean SSWS during 6MWT was significantly higher (*p* = 0.034) when participants used the 1M10 (0.71 ± 0.27 m/s) compared to SACH (0.67 ± 0.30 m/s).

**Figure 5:**
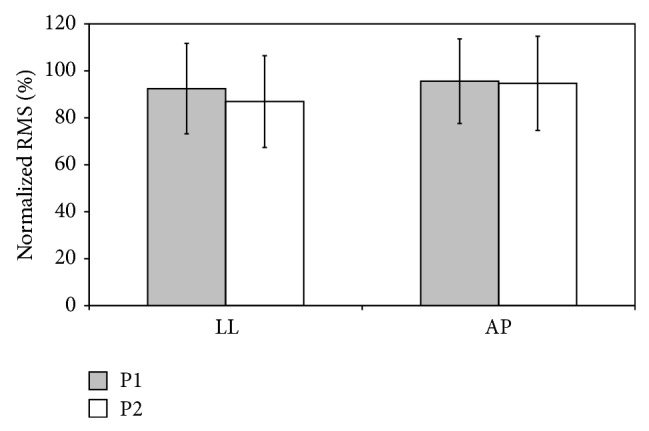
Root Mean Square (RMS) of acceleration along anteroposterior (AP) and laterolateral (LL) axes normalized with respect to those of craniocaudal (RMS-CC) by using the inverse of their percentage ratio in P1 and P2 (mean ± SD).

The UGS results are shown in Figure 5. Despite the higher speed, accelerations on the transverse plane normalized for CC were slightly but not significantly lower, with the 1M10 compared to the SACH foot (LL: 86.94 ± 19.56% versus 92.44 ± 19.26%, *p* = 0.056; AP: 94.67 ± 20.05 versus 95.62 ± 18.00, *p* = 0.785).

## 4. Discussion

The topic of this paper, comparing the clinical effects of non-articulated and multiaxial foot-ankle mechanism on performance of low-activity users, addresses a concept that historically has received little attention in prosthetics research and is of clear interest to the clinical community involved in amputee rehabilitation.

Lower limb amputees must learn to manage their own prosthesis in order to optimize mobility not only relating to the walking performance but also to negotiating ramps and stairs, balance performances, gait stability, and general comfort. The prosthetic componentry may significantly affect these aspects, which are key indicators of the amputee's autonomy in activities of daily living. The collected outcomes' measures were selected in order to investigate these important clinical aspects related to the use of prosthesis, addressing the working hypothesis and the central objective of this study. We have investigated more aspects of the prosthesis usage as overall mobility, balance, and general satisfaction in a group of low-mobility TTAs fitting the conventional SACH foot and, after an adequate acclimation period, a multiaxial one. 1M10 is endowed with a multiaxiality feature without dynamic elements on board and was supposed to yield a comparable or more adaptable gait with respect to the conventional SACH foot in the selected sample.

Comparisons between the two considered prosthetic feet showed that when using the multiaxial foot there were significant improvements in the overall SSWS, HAI, SAI, LCI-5, BBS, and in some PEQ scales.

No significant differences were detected in the UGS, showing a similar clinical response with both prosthetic devices during walking. So, the increased number of DoFs in the multiaxial joint appears to not jeopardize the subjects' stability during locomotion but to improve the perceived safety, as confirmed by the PEQ results (ambulation) and by an improved walking performance during the 6MWT. Indeed the SSWS was significantly higher in our sample when participants used 1M10. Overall the SSWS value obtained in our study with SACH is considerably lower than that of Torburn (1.12 ± 0.26 m/s), Snyder (1.06 ± 0.16 m/s), and Nielsen (1.19 ± 0.28 m/s), but all these authors have enrolled unilateral TTAs with higher level of mobility than our patients [6, 23, 38]. Zmitrewicz et al. [1] did not find significant differences in SSWS, almost equal with SACH and SACH-MA (SACH: 1.04 ± 0.15 m/s; SACH-MA: 1.03 ± 0.15 m/s) and however higher than our values, but also their study included only active TTAs [1]. Moreover the SACH-MA, although it has multiaxial properties, was a prototype designed specifically for the study and not commercially available.

It was not possible to compare our data with those of Marinakis [13], who reported the comparison between SACH and the Greissinger Plus foot because their study did not report the overall SSWS but only walking speed of the limb involved, obtained by dividing the step length over the step time.

The LCI-5 did significantly improve fitting the multiaxial foot. This result was in contrast with those reported by Gailey et al. who stated that the LCI-5 is unable to detect differences in participants' perception of mobility after fitting with four categories of prosthetic feet [39]. It must be considered, however, that the LCI-5 score of SACH users in the study of Gailey et al. was on average above 54 points, which is considerably higher than that of our sample. We have found a median LCI-5 score of 45 with the SACH foot and a score of 49 with the multiaxial one, without any “ceiling effect” but with a significant increase (*p* = 0.012).

SAI and HAI scores and SAI times results showed a significantly improved ability in walking up and down on inclined surfaces and ascending/descending stairs exploiting the multiaxial behavior of the 1M10, as shown in Table 3. SAI and HAI scores improved in P2 by a small but statistically significant quantity. This result was due to the fact that the SAI score was higher using the 1M10 in 6 subjects, equal in the two feet in 14 subjects, but in none of them was the SAI-score result higher using the SACH. Similarly the HAI score was equal in 15 participants and higher in 5 between P1 and P2. Therefore the statistical significance observed in HAI and SAI score results probably derives from the fact that the increase, although very small, is highly systematic. Although they have shown a better gait pattern, confirmed by a systematic improvement in HAI score, participants exhibited a similar duration in walking up and down the ramp. As expected, indeed, the ascent and descent HAI times showed only a slightly improving trend in P2. This result is not surprising because persons with lower limb amputation usually feel insecure on ramps especially walking downhill for fear of their body weight pulling forward, unbalancing them, and, subsequently, leading to a fall. The HAI score measures persons' overall ability and quality of performance during hill negotiation. It is conceivable that subjects in P2 systematically improved the quality of the motor task execution, without affecting the measured HAI uphill/downhill time. In addition, it should be considered that both the feet tested have no active dorsiflexion or dynamic components for greater uphill propulsion.

As shown in Table 2, all TTAs tested had a low risk of falling, both with the SACH and the 1M10. Indeed a BBS score of 0–20 indicates a high risk, 21–40 a medium risk, and 41–56 a low risk of falling [17]. Differences in BBS have been said to be statistically significant, with a score improvement of nearly 10% with the multiaxial foot. However, the 4-point score increase value is very close to the minimum detectable change of this instrument for other pathological groups (e.g., for people with stroke, it is about 5 points [40]).

Usually, higher velocity intrinsically implied higher accelerations. Both raw RMS and normalized ones were investigated during UGS. The RMS along the CC axis is usually more dependent on speed, and in fact it was higher when subjects walked faster using the 1M10. Conversely, when this parameter was used to normalize accelerations along the other two axes, no significant differences were detected. So, according to previous studies on upper body accelerations during gait [32], it is conceivable to assert that subjects walked faster when they used 1M10 compared to when they used the SACH, with similar upright stability between the two feet. This means that stability is not jeopardized by 1M10 use, despite leading to faster SSWS.

Relating to the PEQ results, participants rated abilities to ambulate better when using the 1M10 (ambulation scale), with an improvement of nearly 16% in the score. This fact confirms the results obtained in the outcome measures related to mobility presented above. Also the perceived residual limb health improved in P2 with statistical evidence (*p* < 0.05). One might speculate that this result may be due to lower share and stress forces perceived on the residual limb when the multiaxial foot was used, but this study does not provide any scientific evidence to support this hypothesis. The other scales of the PEQ that were significantly improved, passing from the SACH to the 1M10 foot, were “utility” and “well-being” (*p* < 0.05) of the general comfort and self-rated quality of life. The score of the remaining 5 scales of PEQ showed small improvements, which were not statistically significant. PEQ data are coherent with those of SSWS, LCI-5, and SAI, with greater perceived mobility using the multiaxial foot.

Previous studies that compared two feet did not detect such evident differences [40], but this could be due to the heterogeneous or small samples of the participants involved. In fact studies indicate a lack of consistency in quantitative gait measures in prosthesis users, even with similar populations walking with comparable prosthetic configurations [39]. The biomechanical analysis of the two previous studies on the comparison between SACH and multiaxial foot [1, 13] showed that hypomobile TTAs do benefit from the flexibility provided by multiaxial ankles, improving the spatiotemporal time parameters and the ability to generate impulses with the residual leg and, thus, the loading symmetry between the residual and sound limbs. As reported by the authors [1, 13], their study limitations refer to the fact that they enrolled solely vascular [1] or traumatic (in the early stages of rehabilitation and without the definitive prosthesis) [13] TTAs, focusing exclusively on the biomechanical analysis and not covering major aspects of life that can be affected while living with the prosthesis. Indeed, as highlighted by Marinakis [13], prescribing a prosthetic component as foot only on the basis of gait analysis data is unwise. In the present study we enrolled a sample of 20K1-K2 TTAs, both vascular and traumatic, definitive prosthesis users: so, it is conceivable that these subjects had already internalized the presence of the prosthesis into their locomotor body schema. The contribution of this work provides useful evidence that could help practitioners in the amputee rehabilitation to select the more appropriate prosthetic foot for hypomobile TTAs through the data integration derived from experimental outcome measurements, such as mobility and stability, and clinical self-reported scales, testing the same patients with two different types of feet and overcoming some limitations of previous studies [1, 13]. Our results may be closely correlated with the findings of Zmitrewicz et al. [1]. Indeed, they assert that SACH-MA produced the best combination of residual-to-intact leg braking and propulsive ratios: the clinical improvements can be related to loading symmetry and the ability to generate increased propulsion with the residual leg with the multiaxial feet. Therefore, to have a prosthetic joint that better reproduces the DoFs of the natural ankle (without dynamic and elastic elements that, as is well known, could destabilize the hypomobile amputees' gait) can improve quality of walking, balance, and satisfaction perceived. In light of the results, significant improvements were observed in most of the outcome measures collected using the multiaxial foot. Thus, the working hypothesis was confirmed. Nevertheless, such improvements can be considered more or less clinically relevant. However, it can be argued that the increased number of DoFs in the multiaxial joint can provide a better, comparable but certainly not worse clinical response with respect to the traditional SACH.

### 4.1. Study Limitations and Future Research

The study protocol, despite including walking on slopes, did not use specific functional tests focused on the comparison of the two feet on uneven terrain in which benefits of a multiaxial foot may be still greater than the rigid one on the basis of its design features.

Although the biomechanical analysis on the comparison between SACH and multiaxial foot has already been conducted [1, 13], further studies could thoroughly examine the biomechanical aspects of the 1M10 involving energy cost, kinetic and kinematic analysis and investigating the underlying mechanisms of the relationship between user performance/perception and prosthetic mechanical properties to more definitively explain the clinical effects that we have observed. Without actually quantifying differences in the mechanical function of each prosthesis, it may be argued that 1M10 behaves similarly to the SACH foot when participants are subjected to the conditions associated with the battery of outcome measures collected. Moreover it may be interesting to conduct an EMG analysis to understand how the change of foot could affect the residual muscle activities and therefore explain the significant improvement between P1 and P2 in the “residual limb health” domain of PEQ. In addition a multiaxial foot could be compared with ESAR feet on the same category of patients or, alternatively, on the TTAs with higher mobility level to provide, more accurately, the applicable limit of this device.

## 5. Conclusion

To identify the most proper prosthesis and improve user efficiency and safety, it is important to study the effect of different feet on a specific category of amputees.

This paper fills an important gap in the literature as, to the best of our knowledge, there are no similar studies about the considered prosthetic feet for low-activity users with so wide a range of clinical evaluations. After the replacement of the SACH with a multiaxial foot, patients have maintained the same level of stability and perceived safety, while presenting a significant albeit slight improvement in some important clinical aspects of TTAs' daily living, as overall mobility, balance, general comfort, and the perceived satisfaction with their own prosthesis.

Our findings demonstrate that a multiaxial foot represents an alternative solution with respect to the conventional SACH in the prescription of prosthetic feet for hypomobile TTAs.

Thus, the range of prosthetic devices available to practitioners involved in amputee rehabilitation is increased, therefore allowing them to select the most appropriate solution for each specific subject based on their clinical experience.

## Figures and Tables

**Table 1:** Demographic features of study subjects with different Medical Functional Classification Level (MFCL) fitting two main prosthesis types: Total Surface Bearing (TSB) and Prosthesis Tibiale Kegel (PTK).

Subjects	Gender	MFCL level	Age (years)	Body mass (kg)	Height (cm)	Time since amputation (years)	Prosthesis type	Etiology
1	Male	K2	66	100	171	2	TSB	Vascular disease
2	Male	K2	71	60	172	5	TSB	Neoplasia
3	Female	K2	65	100	159	24	PTK	Trauma
4	Male	K2	51	82	182	34	PTK	Trauma
5	Male	K2	59	80	168	5	TSB	Trauma
6	Male	K2	74	75	165	56	PTK	Trauma
7	Male	K2	68	72	172	8	TSB	Vascular disease
8	Male	K2	74	81	165	8	TSB	Vascular disease
9	Male	K2	67	77	180	5	TSB	Vascular disease
10	Male	K2	65	77	163	5	TSB	Vascular disease
11	Male	K2	68	88	178	4	TSB	Vascular disease
12	Male	K2	58	100	180	4	TSB	Vascular disease
13	Male	K2	63	69	163	5	TSB	Vascular disease
14	Female	K2	74	80	160	7	TSB	Vascular disease
15	Male	K2	63	97	171	3	TSB	Trauma
16	Male	K2	78	56	161	2	TSB	Vascular disease
17	Male	K1	71	70	160	11	TSB	Vascular disease
18	Female	K2	64	60	159	1	TSB	Vascular disease
19	Male	K2	74	74	173	6	TSB	Vascular disease
20	Male	K2	60	72	167	1	TSB	Trauma

Mean			66.7	78.7	168.6	9.8		
SD			6.7	13.2	7.6	13.5		

**Table 2:** Score results of Berg Balance Scale (BBS), Locomotor Capability Index-5 (LCI-5), Stair Assessment Index (SAI), and Hill Assessment Index (HAI) during data collection sessions fitting the SACH (P1) and the multiaxial foot (P2) compared by means of a nonparametric Wilcoxon signed rank test. Data shown in this table are expressed as median (interquartile range); *Z* and *p* values (significant in bold) are reported.

Outcome	Score P1	Score P2	*Z*	*p*
BBS	50.5 (7.5)	54.5 (3.0)	−3.281	**0.001**

LCI-5	45 (18)	49 (16)	−2.521	**0.012**

HAI	7 (4.25)	7 (4.0)	−2.041	**0.041**

SAI	11 (4.25)	11.5 (4.25)	−2.251	**0.024**

**Table 3:** Uphill and downhill times in Hill Assessment Index (HAI) and going up and down times in Stairs Assessment Index (SAI) during the first (P1) and the second (P2) phases of evaluation, compared using two-tailed paired *t*-test. Values are expressed as mean ± standard deviation (SD); *t* and *p* values (significant in bold) are reported.

Outcome	Time (s) P1	Time (s) P2	*t*	*p*
HAI Walking up	43.6 ± 28.4	40.3 ± 33.0	2.064	0.276

HAI Walking down	40.8 ± 25.4	34.5 ± 17.2	1.121	0.053

SAI Ascending	32.0 ± 16.4	27.9 ± 16.4	2.522	**0.021**

SAI Descending	37.1 ± 23.4	31.8 ± 19.9	3.912	**0.001**
